# Large-scale database mining reveals hidden trends and future directions for cancer immunotherapy

**DOI:** 10.1080/2162402X.2018.1444412

**Published:** 2018-03-29

**Authors:** Jakob Nikolas Kather, Anna Sophie Berghoff, Dyke Ferber, Meggy Suarez-Carmona, Constantino Carlos Reyes-Aldasoro, Nektarios A. Valous, Rodrigo Rojas-Moraleda, Dirk Jäger, Niels Halama

**Affiliations:** a Department of Medical Oncology and Internal Medicine VI, National Center for Tumor Diseases, University Hospital Heidelberg, Heidelberg, Germany; b Heidelberg Site, German Cancer Consortium (DKTK), Heidelberg, Germany; c Clinical Cooperation Unit Applied Tumor Immunity, D120, German Cancer Research Center (DKFZ), Heidelberg, Germany; d Clinical Unit for Experimental Oncology Therapy, Thoraxklinik, University of Heidelberg, Heidelberg, Germany; e Department of Electrical and Electronic Engineering, School of Mathematics, Computer Science and Engineering, City, University of London, London, UK

**Keywords:** Cancer immunotherapy, checkpoint inhibition, database mining, gastrointestinal cancer, lung cancer, translational research

## Abstract

Cancer immunotherapy has fundamentally changed the landscape of oncology in recent years and significant resources are invested into immunotherapy research. It is in the interests of researchers and clinicians to identify promising and less promising trends in this field in order to rationally allocate resources. This requires a quantitative large-scale analysis of cancer immunotherapy related databases.

We developed a novel tool for text mining, statistical analysis and data visualization of scientific literature data. We used this tool to analyze 72002 cancer immunotherapy publications and 1469 clinical trials from public databases. All source codes are available under an open access license.

The contribution of specific topics within the cancer immunotherapy field has markedly shifted over the years. We show that the focus is moving from cell-based therapy and vaccination towards checkpoint inhibitors, with these trends reaching statistical significance. Rapidly growing subfields include the combination of chemotherapy with checkpoint blockade. Translational studies have shifted from hematological and skin neoplasms to gastrointestinal and lung cancer and from tumor antigens and angiogenesis to tumor stroma and apoptosis.

This work highlights the importance of unbiased large-scale database mining to assess trends in cancer research and cancer immunotherapy in particular. Researchers, clinicians and funding agencies should be aware of quantitative trends in the immunotherapy field, allocate resources to the most promising areas and find new approaches for currently immature topics.

## Introduction

Cancer immunotherapy is widely regarded as one of the most promising approaches for treating metastatic cancer.^1^ It has been in the focus of basic, translational and clinical research for years and significant resources have been invested in finding new immunotherapy treatments with clinical efficacy.

Anecdotally, most clinicians and researchers in the field are aware that clinical translation has not been equally successful for each subfield over the last years. For example, it is well-known that therapeutic vaccines were intensely investigated and shaped immunotherapy for years but have not yet made a direct clinical impact. Also, immunotherapy quickly reached clinical application in melanoma,^2^ while gastrointestinal cancer types are still lagging behind.^3^ These shifts within the cancer immunotherapy field are highly relevant for clinicians, researchers and funding agencies. However, until now, these changes have not been quantified in a way that allows an unbiased assessment of past and possible future trends.

In the present study, we quantified the development of the cancer immunotherapy field from 1986 to 2017 to reveal previously hidden trends. This type of quantitative and unbiased analysis is of high interest to researchers and clinicians because it can guide the allocation of resources for future research and clinical trials. Specifically, we focused on the comparison of treatment approaches, translational research topics and different tumor entities (organ of the primary tumor, according to the International Statistical Classification of Diseases and Related Health Problems, ICD-10). Among various types of cancer immunotherapy,^4^ we looked at the development of oncolytic viruses,^5^ cell-based therapies,^6^ therapeutic vaccines,^7^ checkpoint inhibitors^8^^,^^9^ as well as chemotherapy and radiation therapy. These treatment types were separately analyzed for all tumor entities in order find out which approaches would be most promising in specific entities in the future. To quantify developments in basic and translational cancer research, we included a wide range of topics such as the combination of immunotherapy with stroma^10^ and cancer-associated fibroblasts,^11^ angiogenesis,^12^ tumor-specific antigens,^13^ neoantigens,^14^ microbiota,^15^ drug resistance,^16^ myeloid cells,^17^ stem cells,^18^ epigenetics,^19^ cell death and autophagy^20^^,^^21^ as well as metabolism.^22^ All trends were analyzed over time, keeping in mind that the field was profoundly changed by landmark events such as the first clinical report of effective checkpoint inhibition in cancer patients in 2003.^23^^,^^24^ Inhibitors of immune receptors and ligands are currently the largest class of approved immunotherapy drugs.^25^^,^^26^ To investigate this subfield in detail, we used a graph-based approach to visualize which of these checkpoint pathways was in the focus of research efforts during the last years. Also, this analysis was used to identify promising combination approaches to target checkpoint signaling pathways.

In short, we present a novel method for data collection, analysis and visualization of changing trends in cancer immunotherapy from 1986 to 2017 and discuss their implications.

## Methods

### Database queries

Based on previous literature reviews and other publicly available resources, we manually curated a list of keywords to enable the comparison of different tumor entities (organ of the primary tumor, e.g. brain, breast, sarcoma, etc., complete list in Suppl. Table 1), treatment approaches (e.g. adoptive cell transfer, oncolytic viruses, checkpoint inhibition, etc., complete list in Suppl. Table 2), translational research topics (e.g. apoptosis, stem cells, epigenetics, etc., complete list in Suppl. Table 3) and cell types (e.g. myeloid, lymphoid, etc., complete list in Suppl. Table 4). Resources for therapeutic agents were the “NIH: A to Z List of Cancer Drugs” (retrieved from https://www.cancer.gov/about-cancer/treatment/drugs on 11 Nov 2017) and all FDA approvals 2016 and 2017 (retrieved from https://www.fda.gov/Drugs/InformationOnDrugs/ApprovedDrugs/ucm279174.htm on 11 Nov 2017). Publication data were automatically mined from MEDLINE, the database of the United States National Library of Medicine (NLM), and its related search engine PubMed (https://pubmed.gov). Furthermore, we analyzed all cancer immunotherapy clinical trials registered in the official US (https://clinicaltrials.gov) database. PubMed articles were identified by the following master search keyword: (“tumor”[All Fields] OR “tumor”[All Fields] OR “neoplasms”[MeSH Terms] OR “neoplasms”[All Fields] OR “cancer”[All Fields]) AND (“immunotherapy”[MeSH Terms] OR “immunotherapy”[All Fields]), in a similar way to a previously published study.^27^ For clinical trials, the master keyword was: “cancer immunotherapy”. For clinical trials, all accessible trial metadata (title, description and structured information) was downloaded from respective databases. All database queries were made in November 2017.

### Data analysis

All data analyzes and visualizations were conducted with self-developed MATLAB scripts (R2017a, MathWorks, Natick, MA, USA). Data were normalized to the number of total immunotherapy articles (or trials, respectively) in each year. Data points were smoothed with a moving average filter (lowpass filter with a coefficient equal to the reciprocal of the time span and a window size of five years). All scripts are released open-source and are available under the following DOI: 10.5281/zenodo.1190620

### Trumpet plot

To illustrate the temporal variation of the incidence of keyword groups, we used the self-developed “*trumpet plot*”. Normalized and smoothed timelines were visualized as the height of a “trumpet” shape in a 2D. In 3D, the diameter of a cylinder represented the normalized number of research items in a given year with time as the vertical axis. Perceptually optimized colour scales from the “Color Brewer” project were used to visualize data.^28^

### Graph-based analysis and network plot

To investigate the degree of connectivity between similar keywords in a specific subfield, we used a graph-based analysis. This was employed for keywords that represented different immune checkpoint molecules e.g. PD-1, PD-L1, CTLA-4, CD80, etc. (full list in Suppl. Table 1). Each keyword was represented by a node which was visualized as a circle. The size and color of the circle depicted the number of research items matching this keyword. The distance between the node and the width of the connecting edge represented the co-occurrence of two keywords. Logarithmic scaling was used for the circle size and the edge width. Isolated nodes without any connection to other nodes were discarded. Low-abundant nodes (< 10 hits) and edges were also discarded.

## Results

### Shift from vaccination to checkpoint inhibition in clinical and translational studies

**Figure 1.**
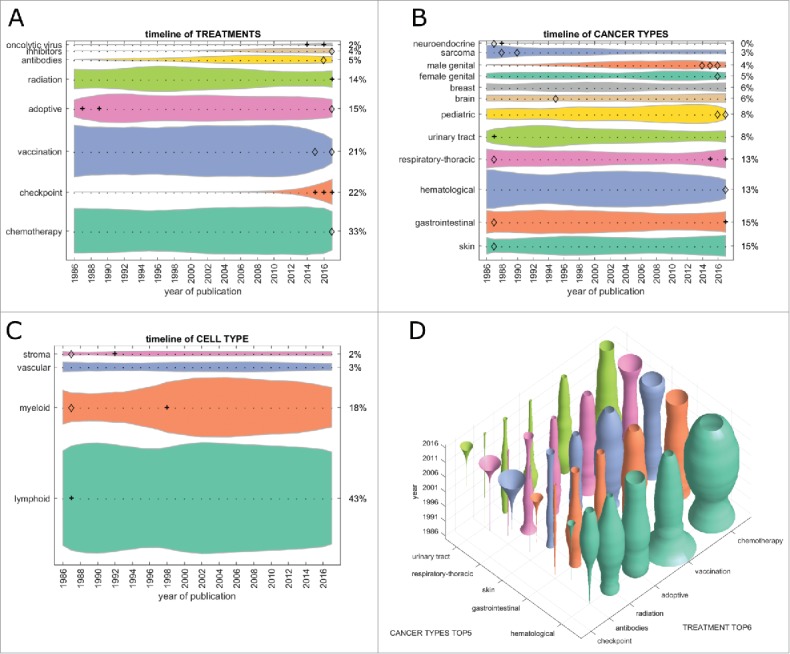
Trends in PubMed publications from 1986 to 2017 by topic. This Fig. summarizes all PubMed listed cancer immunotherapy articles grouped by category. (A) Among all cancer immunotherapy articles published in 2017, 33% referred to one or more specific chemotherapy drugs (bottom shape). This proportion was roughly constant over three decades. Contrariwise, checkpoint inhibition was almost absent before 2010, showing an accelerating growth afterwards. (B) Hematological neoplasms were the most commonly investigated immunotherapy target until 2014, when they were overtaken by gastrointestinal and skin neoplasms. (C) Among all major cell types in the tumor microenvironment, myeloid cells were rapidly gaining interest around the year 2000. Afterwards, no significant change whatsoever was observed. (D) Bivariate analysis of treatment types versus cancer types in PubMed cancer immunotherapy publications. Checkpoint inhibition shows a markedly increasing trend (“trumpet”) in skin, respiratory, but also urinary tract and gastrointestinal cancer. (A-C) + significant rise of growth rate within one year (anomaly >95%), ◊ significant decrease of one-year growth rate (anomaly < 5%).

**Figure 2.**
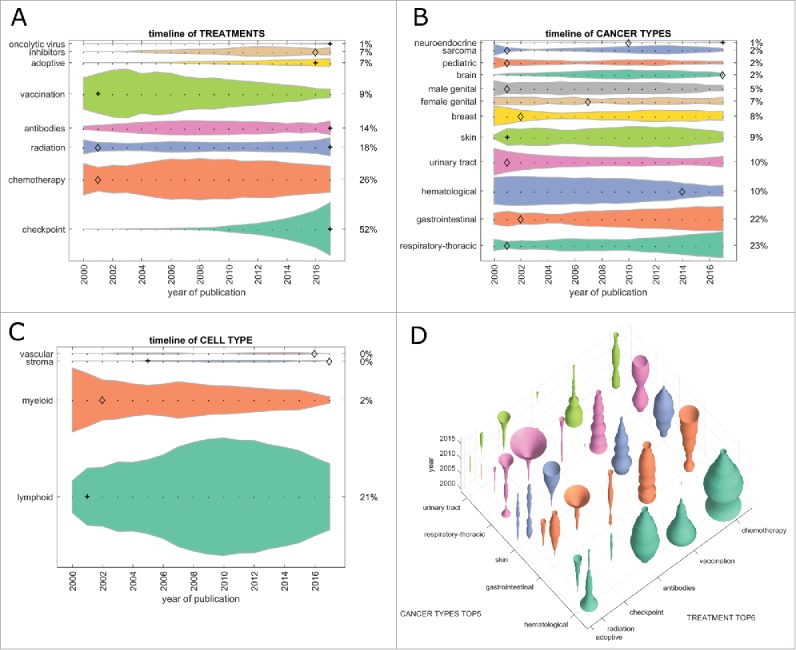
Trends in clinical trials from 2000 to 2017 per topic. This Fig. summarizes all registered clinical trials of cancer immunotherapy grouped by category. (A) Immune checkpoint inhibition has rapidly become the most common therapy approach between 2010 and 2017. At the same time, vaccination approaches have greatly diminished, being subject to only 9% of clinical immunotherapy trials in 2017. (B) As in PubMed publications, hematological neoplasms have markedly lost ground, yielding to gastrointestinal and respiratory neoplasms in recent years. (C) Among all major cell types in the tumor microenvironment, myeloid cells were in the focus of research interest around 2000, diminishing afterwards and only being investigated in 2% of immunotherapy clinical trials in 2017. (D) Bivariate plot of treatment types versus cancer types in cancer immunotherapy clinical trials. Checkpoint inhibition shows an increasing trend (“trumpet”) in respiratory and gastrointestinal cancer. (A-C) + significant rise of growth rate within one year (anomaly >95%), ◊ significant decrease of one-year growth rate (anomaly < 5%).

First, we analyzed the contribution of major treatment types to the cancer immunotherapy literature. In the PubMed database, chemotherapy was the most frequent treatment that articles could be matched to (33% in 2017, Fig. 1A). Checkpoint inhibition grew significantly (indicated by a **+** in the graphs) from 2015 and was the second most abundant treatment type in 2017. Therapeutic vaccination as a form of cancer immunotherapy dropped from position 1 to position 3 in 2017, with significant decrease (indicated by a diamond in the graphs) between 2015 and 2017. These trends were even more pronounced in clinical trials where checkpoint inhibition was matched in more than 50% of all items in 2017, chemotherapy being second with 26% and vaccination steadily dropping to only 9% of clinical trials in 2017 (Fig. 2A). Adoptive cell-based therapies (including chimeric antigen receptor [CAR] T-cells) contributed to 15% of all research items in 2017 and to 7% of all clinical trials (Fig. 1A and Fig. 2A).

### Lung and gastrointestinal cancer as prime targets for immunotherapy

Next, we analyzed cancer immunotherapy research efforts for each tumor entity. In articles indexed in PubMed, hematological neoplasias (hema.) were the prime immunotherapy target until 2015/2016, but have decreased significantly since, yielding to skin and gastrointestinal (GI) neoplasms (Fig. 1B). Among the top five tumor entities (skin, GI, hema., respiratory-thoracic [lung] and urinary tract), only lung and GI showed a significant growth in the last five years (Fig. 1B). This pattern matched clinical trial data (Fig. 2B) where lung and GI tumors were the top two cancer entities by far. Again, hematological neoplasms rapidly (and in one year significantly) decreased in importance; also, sarcoma continuously decreased in importance over the years (Fig. 2B).

Subsequently, we asked how the different therapy approaches were reflected in each major tumor entity. In the research literature, checkpoint inhibitors have increased in importance in the last five years in all top five tumor entities (Fig. 1D). The reverse trend can be observed in vaccination and chemotherapy, although these still have a large presence. Much more pronounced effects were observed in clinical trials (Fig. 2D): Here, lung and GI neoplasms were the two most dynamically growing field with growth in skin cancer reaching a plateau and hematological neoplasms vanishing almost completely.

### A transient 1990s interest in myeloid cells left no trace in the clinic

Cancer immunotherapy aims to (re)invigorate the host immune response against malignant cells and all types of cancer immunotherapy use cells in the tumor microenvironment as their effectors. We analyzed the quantitative contribution of cell types in the immunotherapy literature. Items related to myeloid cells significantly increased its presence in PubMed in the late 1990s (Fig. 1C), matching a large contribution to clinical trials at that time (Fig. 2C). However, this transient interest in myeloid cells plateaued in the scientific literature and rapidly decreased in clinical trials. Not surprisingly, lymphoid cells were the largest single group of cells in 2017 in scientific publications and clinical trials.

### Revival of radiation and chemo-immunotherapy

Having analyzed major trends among treatment types, cancer types and cell types, we looked for non-obvious trends in the dataset. We found that among treatment types, radiation was only at position five in scientific articles (Fig. 1A) but at position three in clinical trials (Fig. 2A). In both cases, the growth rate in 2017 significantly exceeded that of previous years. These trends followed a decrease during the early 2000s in radiation therapy in articles and clinical trials (Fig. 1A and Fig. 2A). Based on these data, we conclude that we are currently witnessing a revival of the use of radiation in cancer immunotherapy.

**Figure 3.**
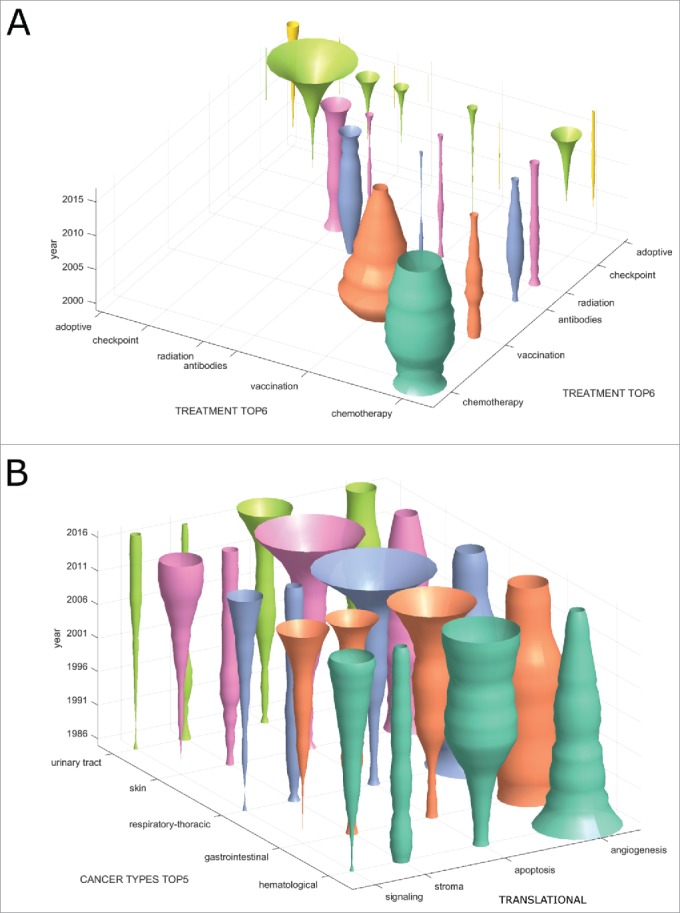
Emerging immunotherapy paradigms. (A) Co-occurrence of cancer immunotherapy treatment approaches in clinical trials between 2000 and 2017. On the diagonal, the development of individual treatment approaches is shown with checkpoint inhibition displaying a rapid increase. Off the diagonal, treatment combinations are shown with chemotherapy and checkpoint inhibition being the most common and rapidly growing combination. (B) This bivariate plot shows cancer immunotherapy trends grouped by translational research topics and major cancer types based on all PubMed publications between 1986 and 2017. Among signalling, stroma, apoptosis and angiogenesis, apoptosis is the most rapidly growing topic in all major cancer entities except hematological neoplasms. Stroma and signalling are most rapidly increasing in gastrointestinal cancer.

We hypothesized that other non-obvious trends might be hidden in treatment combinations and therefore analyzed co-occurrence of treatment types in clinical trials (Fig. 3A). In this analysis, the diagonal of the matrix corresponds to Fig. 2A. We found that the only markedly increasing treatment combination is chemotherapy plus checkpoint inhibition (Fig. 3A). In contrast, virtually no registered clinical trials investigate the combinations vaccination plus checkpoint inhibition or adoptive cellular therapy plus checkpoint inhibition.

### Stroma and apoptosis in gastrointestinal cancer

Our automatic approach for database mining allowed for an analysis of translational research topics per tumor type. For clarity, only a part of this analysis is shown in Fig. 3B. We found that among translational research topics in immunotherapy articles, angiogenesis is decreasing in importance in all major cancer entities. In contrast, apoptosis (and other forms of cell death as well as autophagy) is rapidly gaining ground in GI, lung and skin cancer (Fig. 3B). Interestingly, the quantitative contribution of cancer stroma to immunotherapy articles is stagnating or decreasing in all major cancer entities except GI cancer (Fig. 3B). Complementing our above-described finding that GI cancer is one of the most dynamically growing research topics in immunotherapy, we conclude that especially apoptosis and stroma are promising subfields in this entity.

### Translational activities vary considerably between tumor types

**Figure 4.**
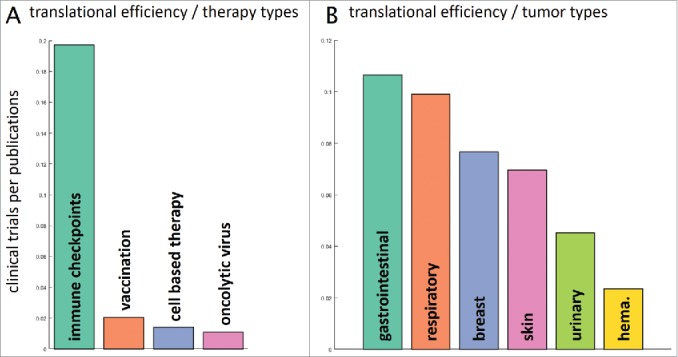
Translational efficiency. We asked how the number of research publications influences the number of clinical trials in subsequent years. To this end, we analyzed PubMed articles for specific fields in a five-year period (2006–2011) and evaluated the number of matching US-registered clinical trials in the following five years (2012–2016). This yields a measure of translational efficiency (clinical trials per research publication). (A) Among therapy types, immune checkpoint inhibitors had the highest translational efficiency with approximately 0.2 trials per publication. Scientific findings in vaccination and cell-based therapy were not efficiently translated to the clinic. (B) Among major tumor entities, translational efficiency was highest for gastrointestinal tumors and lowest for hematological and lymphoid malignancies (hema.). It is in the interest of the research community to increase translational efficiency in these low-performing fields.

Our next step was to examine the following question: how were preclinical research efforts, measured by the number of indexed items on PubMed, translated into clinical trials? To give a specific answer for all therapy types and major cancer entities, we compared timelines for multiple keywords in PubMed and clinical trial databases. We analyzed the number of clinical trials in the last five years (2012–2016) and normalized these numbers to the respective number of PubMed research items in the preceding five years. Among all therapy types, immune checkpoint inhibition stood out in terms of translational efficiency with close to 0.2 clinical trials per research paper in the reference periods (Fig. 4A). Looking at various tumor entities, the differences in translational efficiency were not as large (Fig. 4B). Highest translational efficiency was visible in immunotherapy of gastrointestinal and respiratory neoplasms while a low translational efficiency was seen in hematological malignancies with just 0.02 clinical trials per article (Fig. 4B).

**Figure 5.**
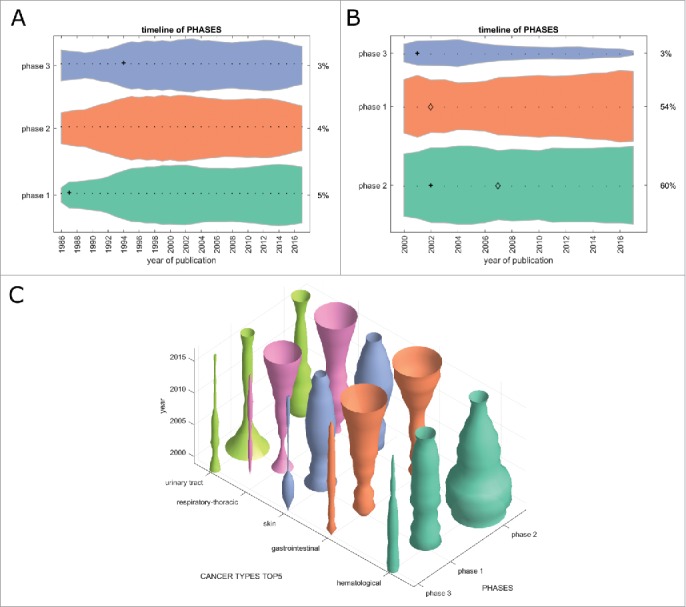
Clinical trial phases. This Fig. shows the development of clinical trials in phase 1/2/3 over time. (A) PubMed articles matching any clinical phase. Only a fraction of PubMed listed articles can be matched to a clinical phase and the proportions between the phases have not changed significantly in the last 20 years. (B) Clinical trials matching any clinical phase, ordered by group size, from bottom to top: phase 2, phase 1, phase 3. Some trials could be matched to multiple phases so that the percentages in 2017 do not necessarily add up to 100%. Phase 2 trials are most abundant and phase 1 trials are slowly growing, albeit not significantly. (C) In stark contrast to the slow overall growth dynamic of clinical trials in the above panels, this panel shows marked changes in clinical trials per cancer entity over time. In gastrointestinal cancer and respiratory-thoracic cancers, phase 1 and 2 trials are currently showing pronounced increase. (A+B) + significant rise of growth rate within one year (anomaly >95%), ◊ significant decrease of one-year growth rate (anomaly < 5%).

Another way of comparing the translational efficiency of immunotherapy subfields is to look at the development of clinical phase 1/2/3 trials over time. We matched all cancer immunotherapy trials registered at clinicaltrials.gov and all PubMed articles (when applicable) to one or more clinical phases. In the timelines in Fig. 5A, a small and stable percentage of PubMed articles can be matched to any clinical trial phase over time. Within registered clinical trials (Fig. 5B), phase 1 and 2 trials are slowly increasing with phase 3 trials decreasing at the same time. However, in general, no pronounced trends were visible in this analysis. This picture changed markedly when analyzing clinical trials for each major tumor entity (Fig. 5C): Phase 1 and 2 trials were rapidly increasing in gastrointestinal and lung cancer in the last five to ten years, but not in other major tumor entities. These data match our above-mentioned finding that GI and lung cancer are the most translationally active fields as compared to skin cancer, hematological neoplasias and other major cancer types.

### Immune-checkpoint networks

**Figure 6.**
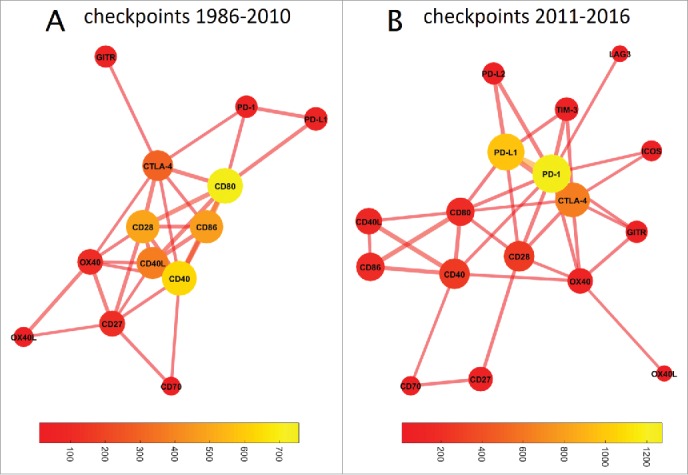
Graph-based analysis of immune checkpoints. In these graphs, the distance between two nodes denotes the co-occurrence while the color of the bubble denotes the frequency of occurrence (bubble sizes are log occurrence). (A) Before 2011, a cluster around CD80/CD86/CD40/CD28 dominated immune checkpoint research. (B) This has fundamentally changed since 2011: The field is now dominated by PD-1/PDL1, with CTLA-4 as a bystander. The number of relevant immune checkpoints has markedly increased. CD80 still occupies a central position in the network, linking the CD40/CD86/CD40L cluster with PD1/PD-L1/CTLA-4. TIM3 and OX40 have also moved closer to the network's core, indicating an increasing importance despite few absolute hits.

Based on above-described results we concluded that checkpoint inhibition makes the largest quantitative contribution to research papers and clinical trials in immunotherapy research and is also the most efficient subfield in terms of clinical translation. Therefore, we performed a more specific analysis and asked how the contribution and intertwining of immune checkpoint molecules and drugs developed over time. Based on our timeline analysis (Fig. 1A) we estimated that around 2011, the increase in checkpoint inhibition publications started. We therefore used the following time frames, 1986–2010 and 2011–2016, to compare co-occurrence of checkpoint molecules in PubMed articles. These comparisons are shown in Fig. 6 as network plots. In 1986 to 2010, CD80 had the highest prevalence (Fig. 6A) and a cluster around CD80/CD86/CD28/CD40L/CD40 dominated the immune checkpoint landscape in PubMed articles. In 2011 to 2016, a marked change was evident and PD-1/PD-L1, which were previously in the periphery of the network, and CTLA-4, which remained in the center, made by far the largest contribution (Fig. 6B). Interestingly, CD80 (B7–1) still occupied a central “hub” position, linking two distant parts of the network with each other.

### Discussion

Tumor immunotherapy research is a dynamically evolving field and has undergone profound changes in the last three decades. While these developments might be implicitly known by researchers who have been deeply involved in the field for a long time, they are probably not apparent to most clinicians and scientists who are now confronted with immunotherapy. Moreover, researchers and clinicians working in the field may have cognitive biases and therefore may not be aware of well and poorly performing subfields of immunotherapy research. In this paper, we presented a quantitative, objective and comprehensive analysis of the changes in tumor immunotherapy research over time which can serve as a rational basis for further discussions.

Skin cancer (mainly melanoma) was the first tumor entity to have effective immunotherapy agents approved and is still in the focus of research papers. Yet, clinical trials now focus on gastrointestinal and respiratory cancers, two major disease classes associated with significant morbidity and mortality. Translational research means that new knowledge should be effectively transferred to the clinic.^29^ Researchers pursuing translational research will therefore meet this aim more easily in an area where translation has been shown to be feasible. By extrapolating these current trends, translational research efforts would be most fruitful in gastrointestinal and respiratory cancer.

As a word of caution, we should also acknowledge that many unexpected breakthroughs come from previously unnoticed areas in biomedical research. Also, not all ongoing research efforts might be reflected by PubMed publications or registered clinical trials. Yet, for the tedious process of using research results from the laboratory to improve treatments in the clinic, a structured and objective projection of future trends can be very useful. Our data-driven analytics approach provides a starting basis for such endeavors.

## Supplementary Material

2018ONCOIMM0085-s02.docx
