# Supplementary material for: Large-scale database mining reveals hidden trends and future directions for cancer immunotherapy
Source: Oncoimmunology. 2018 Mar 29;7(7):e1444412. doi: 10.1080/2162402X.2018.1444412 (PMC5993505; doi:10.1080/2162402X.2018.1444412)
Supplement: 2018ONCOIMM0085-s02.docx [file koni-07-07-1444412-s001.docx]

Large-scale database mining reveals hidden trends and
future directions for cancer immunotherapy

Jakob Nikolas Kather^1,2,3^, Anna Sophie Berghoff^1,4^, Dyke Ferber^1,3^, Meggy Suarez-Carmona^1,3^, Constantino Carlos Reyes-Aldasoro^5^, Nektarios A. Valous^1,3^, Rodrigo Rojas-Moraleda^1,3^,
Dirk Jäger^1,2,3^, Niels Halama^1,2,3^

^1^ Department of Medical Oncology and Internal Medicine VI, National Center for Tumour Diseases, University Hospital Heidelberg, Heidelberg, Germany

^2^ German Cancer Consortium (DKTK), Heidelberg, Germany

^3^ Clinical Cooperation Unit Applied Tumour Immunity, German Cancer Research Center (DKFZ), Heidelberg, Germany

^4^ Clinical Unit for Experimental Oncology Therapy, Thoraxklinik, University of Heidelberg, Heidelberg, Germany

^5^ School of Mathematics, Computer Science and Engineering, City, University of London, London, UK

# - Supplementary Data -

| brain | brain tumour, glioma, central nervous system, glioblastoma, astrocytoma, oligoastrocytoma, oligodendroglioma, ependymoma |
| --- | --- |
| breast | mammary, breast |
| female genital | ovarian, uterine, ovary, endometrial, placenta, uterus, vagina, cervix uteri, vulva, endometrium, corpus uteri |
| gastrointestinal | liver, colourectal, colon, pancreatic, hepatocellular, gastric, stomach, GI tract, pancreas, esophageal, rectal, intrahepatic, biliary, anus, esophagus, oesophagus, small intestine, bile duct, rectum, gallbladder, oesophageal, ileum, anal canal, duodenum, gastroesophageal junction, jejunum, upper GI, lower GI, cholangio, rectosigmoid junction |
| haematological | leukemia, lymphoma, plasma cell, haematological, myelodysplastic syndrome, haematological |
| male genital | prostatic, prostate, testis, penile, penis, seminoma |
| neuroendocrine | neuroendocrine, carcinoid |
| pediatric | adolescent, pediatric, paediatric, neuroblastoma, childhood, medulloblastoma, medullablastoma |
| respiratory-  thoracic | lung, NSCLC, thymus, thymic, thymoma, SCLC, larynx, nasal cavity, trachea, bronchus, middle ear |
| sarcoma | sarcoma, mesothelioma, GIST |
| skin | melanoma, merkel cell |
| urinary tract | renal, kidney, bladder, urothelial, urethra, ureter |

Suppl. Table 1: Keywords for cancer types. Research items matching a specific group of tumours were identified by non-overlapping sets of keywords. Tumour groups are sorted alphabetically, keywords are sorted by occurrence within a group.

| adoptive | adoptive cell, adoptive cellular, t cell transplant, chimeric antigen receptor, chimeric antigen-receptor, car t-cell, car-t cell, lymphodepletion, tisagenlecleucel, axicabtagene ciloleucel, car t cell |
| --- | --- |
| angiogenesis | anti-vegf, bevacizumab, ramucirumab, anti-vegfr |
| antibodies | rituximab, bispecific antibody, trastuzumab, ibritumomab, alemtuzumab, cetuximab, tositumomab, antibody-drug conjugate, gemtuzumab, brentuximab, blinatumomab, panitumumab, ofatumumab, durvalumab, dinutuximab, denosumab, daratumumab, pertuzumab, obinutuzumab, inotuzumab, elotuzumab, ado-trastuzumab, siltuximab, antibody-toxin conjugate, necitumumab, olaratumab |
| checkpoint | checkpoint blockade, ipilimumab, nivolumab, anti-pd-1, lambrolizumab, pembrolizumab, checkpoint inhibition, anti-ctla-4, anti-pd-l1, anti-pd1, anti-pdl1, atezolizumab, anti-ctla4, tremelimumab, avelumab |
| chemotherapy | chemotherapy, cyclophosphamide, fludarabine, busulfan, doxorubicin, melphalan, daunorubicin, methotrexate, cisplatin, fluorouracil, etoposide, vincristine, cytarabine, gemcitabine, dacarbazine, mitomycin, paclitaxel, docetaxel, thalidomide, carboplatin, carmustine, temozolomide, thiotepa, cytostatic, azacitidine, mercaptopurine, oxaliplatin, mitoxantrone, vinblastine, bleomycin, decitabine, epirubicin, irinotecan, ifosfamide, cabazitaxel, idarubicin, capecitabine, bendamustine, chlorambucil, cladribine, hydroxyurea, lomustine, vinorelbine, topotecan, pemetrexed, clofarabine, procarbazine, dactinomycin, thioguanine, mechlorethamine, flutamide, ixabepilone, trabectedin, valrubicin, eribulin, midostaurin, pralatrexate, nelarabine, tipiracil, trifluridine |
| inhibitors | bortezomib, imatinib, sorafenib, sirolimus, lenalidomide, sunitinib, vemurafenib, everolimus, dabrafenib, temsirolimus, trametinib, ibrutinib, erlotinib, pazopanib, dasatinib, gefitinib, cabozantinib, axitinib, idelalisib, vorinostat, lapatinib, nilotinib, crizotinib, venetoclax, pomalidomide, regorafenib, afatinib, carfilzomib, olaparib, aflibercept, cobimetinib, panobinostat, lenvatinib, romidepsin, ruxolitinib, vismodegib, ceritinib, palbociclib, vandetanib, neratinib, alectinib, osimertinib, ponatinib, abemaciclib, acalabrutinib, ixazomib, niraparib, sonidegib, bosutinib, brigatinib, copanlisib, enasidenib, ribociclib, rucaparib, belinostat |
| oncolytic virus | oncolytic virus, oncolytic adenovirus, talimogene laherparepvec, oncolytic hsv, oncolytic measles |
| radiation | irradiated, irradiation, radiotherapy, radiation, radioactive, radiobiology, brachytherapy, radiosensitive, radioligand |
| vaccination | immunization, immunisation, vaccine, vaccination, sipuleucel |

Suppl. Table 2: Keywords for treatment types. Research items matching a specific treatment type were identified by non-overlapping sets of keywords. Groups are sorted alphabetically, keywords are sorted by occurrence within a group.

| angiogenesis | lymphatics, vascular, endothelial, angiogenesis, VEGF, neoangiogenesis, VEGFR, anti-angiogenic, blood-brain barrier, lymphatic vessel, angiopoietin, vasculogenesis |
| --- | --- |
| antigens | Tumour-specific antigen, Differentiation antigen, Epitope, Viral antigen, Overexpressed antigen, gp100 antigen, MelanA antigen, MART-1 antigen, tyrosinase antigen, ny-eso-1 antigen, mage-3 antigen, neoantigen, mage-1 antigen, prame antigen, trp-2 antigen, caspase-8 antigen, bage antigen, beta-catenin antigen, trp-1 antigen, neoepitope, neo-antigen, gage-1 antigen, rage antigen, neo-epitope, sart-1 antigen, p15 antigen, MUM-1 antigen, gage-2 antigen, MAGE-type antigen, b-catenin antigen, hpve7 antigen, cdk-4 antigen, kiaa0205 antigen |
| apoptosis | cell death, apoptosis, apoptotic, FAS, caspase, BCL-2, Survivin, autophagy, antiapoptotic, proapoptotic, BCL-XL, Cytochrome C, XIAP, FADD, SMAC, necroptosis, MCL1, cIAP1, cIAP2, Apoptosome, IAP antagonist, NAIP, Apollon, ILP2, MLIAP |
| drug resistance | treatment resistance, drug resistance, ABC transporter, p-Glycoprotein, efflux pump |
| epigenetics | epigenetic, histone, histone deacetylase, epigenetics, DNA methyltransferase, HDAC, epigenomics, DNMT |
| evolution | evolution, antigen spread, antigen spreading, epitope spreading, epitope spread |
| genetics | genetics, genome, telomerase, telomere, PARP, mutational load, mutation load, hypermutation, mutanome |
| metabolic | metabolism, metabolic, hypoxic, hypoxia, HIF, glycolysis, glycolytic, aerobic, anaerobic |
| signalling | signalling, signalling |
| stem cell | stem cell |
| stroma | TGF-beta, stromal, fibrosis, extracellular matrix, collagen, TGFB1, CXCL12, FAP alpha, matrix remodeling, acta2, aldh1a3 |

Suppl. Table 3: Keywords for translational topics. Research items matching a specific translational research topic were identified by non-overlapping sets of keywords. Groups are sorted alphabetically, keywords are sorted by occurrence within a group.

| lymphoid | lymphocyte, lymphocytes, T cell, T-cell, NK cell, natural killer cell, B cell, B-cell, regulatory T cell, regulatory T-cell, Treg |
| --- | --- |
| myeloid | dendritic cell, myeloid cells, macrophage, granulocyte, macrophages, neutrophil, myeloid suppressor cells, myeloid-derived suppressor cells, mast cell, eosinophil, microglia, basophil |
| stroma | fibroblast, fibroblasts, stroma, CAF, myofibroblast, pancreatic stellate cell |
| vascular | endothelial cell, lymphatic, vasculature, blood vessel, endothelium, pericyte |

Suppl. Table 4: Keywords for cell types in the tumour microenvironment. Research items matching a specific cell type were identified by non-overlapping sets of keywords. Groups are sorted alphabetically, keywords are sorted by occurrence within a group.
